# The Missouri Dental Journal

**Published:** 1884-01

**Authors:** 


### THE MISSOURI DENTAL JOURNAL.

We regret exceedingly to learn that the publication of this old and valuable journal has been suspended. It has had a long and honorable record. For fifteen years it has been active in the spread of dental intelligence, and in its pages have appeared very many articles which have secured a permanent place in our literature. It contains the record of some of the most valuable life-work of Eames, and Chase, and Spalding, and others, and it is a reflection upon western dentistry that it should lack the support necessary to its existence.

A year ago it was removed from St. Louis to Kansas City, and we noted with pleasure that the new editor and his associates seemed to breathe a new life into it, and we hoped that its merits would have been better appreciated. But the publisher was not a practising dentist, and viewed dental interests from a mercantile standpoint, declining to sacrifice himself for any professional good, and hence the old journal has succumbed. Dr. Pearson, assisted by Drs. Patterson and Hungerford, has done good work as a journalist, although he has struggled in the face of many discouragements. We hope that the pens of neither of the editors will be allowed to lie fallow, but that they will still continue active in a field in which they have shown that they can so successfully labor.

There is a lesson to the profession in this death of one of our too few respectable journals. If we are to have a profession at all, it must have a literature. We have too many illiterate men among us, too few who are faithful supporters of the best interests of dentistry, to allow professional journalism to be pecuniarily profitable. The work that is done in this department must be mainly a labor of love. Yet no intelligent man will deny that the journals add greatly to the dignity of dentistry, and are among the most potent influences for the elevation of the professional standard. Without schools and journals our calling would sink to the level of mere handicrafture. Every practicing dentist owes to the journals much of the respect which his avocation receives from the world, and there is not one who is not pecuniarily and directly benfited by the labors of those who have for years toiled unselfishly and without pay, for the good of the body of men with whom they have cast their lot. Thousands of dentists have benefited by these labors, who have either not realized what others were trying to do for them, or seeing it were content to reap the harvests and ignore those who had assisted at the planting. Many a man has, like the lamented McQuillen, toiled for others all his life, only to die with the debt unacknowledged at last. There are so many who take only the bread-and-butter view of their calling, and who, like the maelstrom suck up everything within their reach, rendering nothing back again. They have nothing but sneers for him who devotes his energies to other's good, though they are ready enough to receive all he has to give. If dentistry is to reach its highest capabilities its literature must be supported, and practitioners must realize that they owe an honest debt to those who are working for their good.

We did not set out to write a Jeremiad, though the text was pregnant with useful lessons. Dental journalism has its bright as well as its dark side, and there are many men whose appreciation makes an otherwise oppressive labor, light. The experience of the publishers of this journal is too brief for them to obtrude their own personality into the discussion of such a subject, and yet they could a tale unfold of cheering words, of proffered assistance, of kindly recognition, such as warms the cockles of one's heart, and demonstrates that there is such a thing as brotherhood among men, and unselfish appreciation of what others are trying hard to accomplish. It was the record of such in our December number, that made the editor of another struggling journal cry out in a private letter, "Oh, for a thousand Darbys."

